# Inlay total shoulder arthroplasty for primary glenohumeral arthritis

**DOI:** 10.1016/j.jseint.2021.07.014

**Published:** 2021-09-15

**Authors:** John W. Uribe, John E. Zvijac, David A. Porter, Anshul Saxena, Luis A. Vargas

**Affiliations:** Miami Orthopedics & Sports Medicine Institute, Coral Gables, FL, USA

**Keywords:** Anatomic total shoulder arthroplasty, glenohumeral arthritis, non-spherical humeral head, inlay glenoid, minimal clinically important difference, substantial clinical benefit

## Abstract

**Background:**

Anatomic total shoulder arthroplasty with a nonspherical humeral head and inlay glenoid replacement has been introduced in the past; however, clinical evidence remains limited. We hypothesized that patients with advanced glenohumeral arthritis demonstrate significant improvements in pain and function.

**Methods:**

Prospective patient-reported outcomes (PROs) included the American Shoulder and Elbow Surgeons score, a pain visual analog scale, and satisfaction. Range of motion was compared to the preoperative status. A sensitivity analysis examined responder rates and compared them to literature thresholds using the minimal clinically important difference and substantial clinical benefit. The preoperative glenoid morphology was determined using the Walsh classification. Zone-specific periprosthetic radiolucent lines were quantified at the last follow-up.

**Results:**

Thirty-nine shoulders in 36 patients (3 bilateral) with a mean age of 65.9 years (26 males, 13 females) and a mean follow-up of 41.0 months were included. Ninety-three percent had grade III osteoarthritis, and 7% grade II. The glenoid Walsh classification included A1 (25%), A2 (25%), B1 (22%), B2 (25%), and C (3%). All PROs improved significantly (*P* < .001) with a mean American Shoulder and Elbow Surgeons score from 30.4 to 77.1, a pain visual analog scale from 8.1 to 1.5, and excellent (9.1/10) patient satisfaction. PRO-related responder rates for minimal clinically important difference and substantial clinical benefit were ≥85%. Forward elevation improved from 107° to 155°, and external rotation from 22° to 51°. One intraoperative glenoid rim fracture led to advanced radiolucency; no other clinically relevant lucency was observed.

**Conclusion:**

Treatment with inlay total shoulder arthroplasty demonstrated significant functional improvement, excellent pain relief, and patient satisfaction in patients with advanced shoulder arthritis and various glenoid morphology types. Our initial results provide further support for this new option in primary shoulder replacement.

Inlay total shoulder arthroplasty (iTSA) is a new treatment option for primary shoulder reconstruction in patients with advanced glenohumeral arthritis. Compared to stemless, stemmed, or reverse TSA, bone stock in the proximal humerus is preserved, the nonspherical shape is maintained, and the glenoid joint line is not lateralized. The implant combination aims for anatomic reapproximation on both sides of the joint. Results from partial humeral inlay arthroplasty have been reported in the past.^25^^,^^26^ However, to the best of our knowledge, evidence on the combination of nonspherical humeral head (HH) and inlay glenoid replacement is limited to two recent studies which found significant improvement in patient-reported outcome (PRO) and range of motion (ROM), a high rate of return to work and sport,^4^ and similar results for both concentric and nonconcentric shoulders.^6^

The purpose of this investigation was to report our initial outcomes in patients with a minimum follow-up duration of two years. We hypothesized that the results would demonstrate significant functional improvement and pain relief.

## Material and methods

The study was designed as a prospective, observational study of patients treated with TSA at a single institution. The study protocol (#11-046) was approved by the institutional review board. All patients consented before their participation, and no direct funding was provided for this study. Inclusion criteria consisted of patients older than 18 years with moderate to severe primary glenohumeral osteoarthritis according to the Samilson-Prieto Classification^20^ and clinical symptoms refractory to conservative treatment. Exclusion criteria included proximal humeral bone deficiencies that could jeopardize humeral component fixation and glenoid vault deficiencies that would not accommodate inlay component placement. No restrictions on selection criteria were placed based on the preoperative glenoid Walch classification.^29^

All procedures were performed by two high-volume shoulder arthroplasty surgeons (J.W.U., J.E.Z.) at our institution. The same surgical technique and postoperative rehabilitation protocol were used in all patients. PROs were collected preoperatively, at 4 to 6 weeks, and at 3, 6, 12, and 24 months postoperatively, as well as annually thereafter.

### Inlay total shoulder arthroplasty

**Figure 1:**
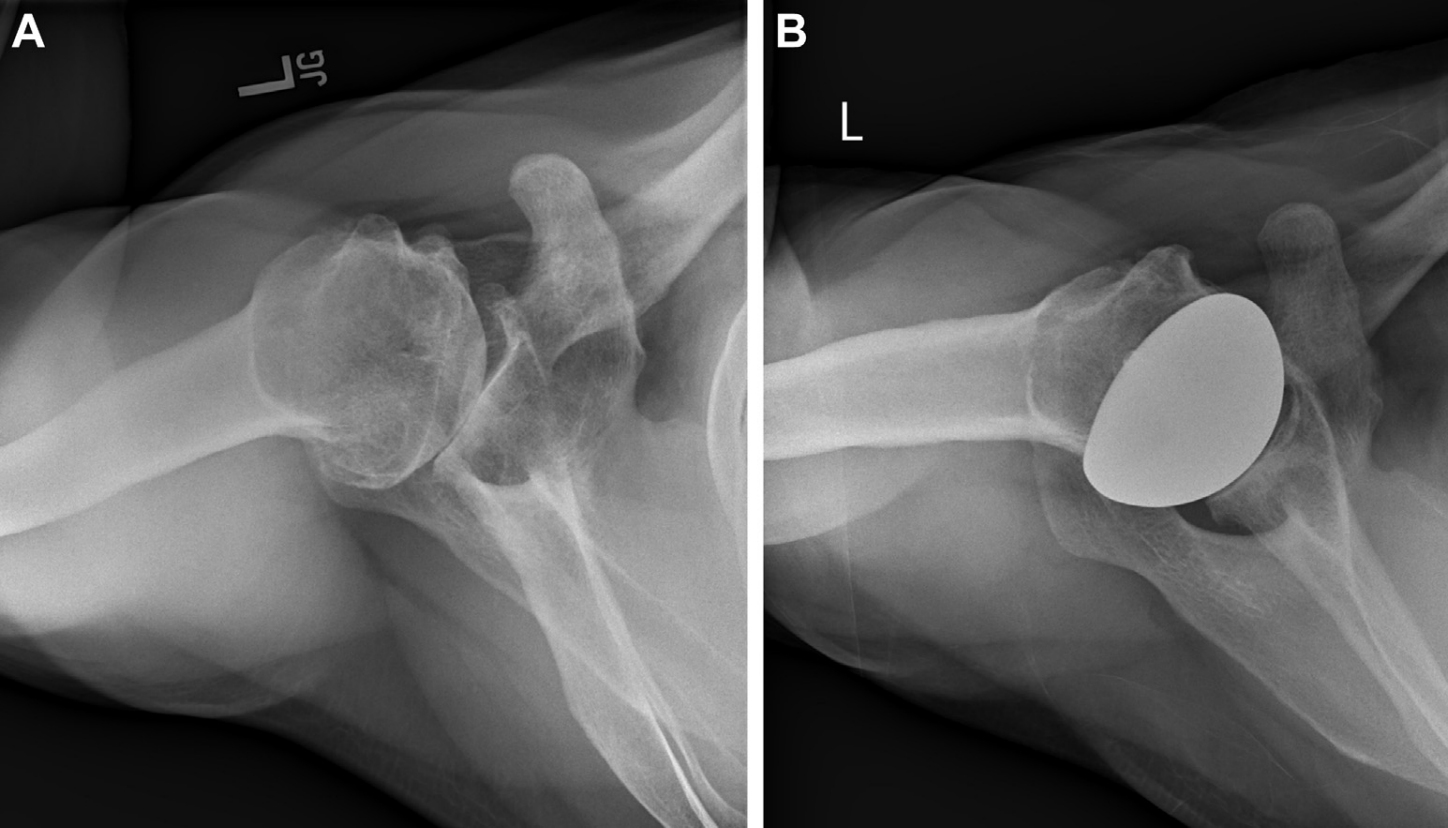
(**A**) Preoperative radiographic imaging. (**B**) Postoperative radiographic imaging.

All implants used in this study (OVO Primary Stemless Total Shoulder System; Arthrosurface, Franklin, MA, USA) included a nonspherical HH, a threaded humeral fixation component, coupled with an inlay glenoid component (Fig. 1 *A* and *B*). Seven HH sizes ranged from 46 mm to 58 mm, each with a 4-mm mismatch between the larger superior-inferior and smaller anterior-posterior (AP) radius of curvature. Glenoid options included 20 mm or 25 mm diameter.

### Surgical technique

With the patient in the beach chair position, a standard deltopectoral approach was carried out as previously described.^26^ Implant templates were used to measure the AP and superior-inferior curves of the HH at its largest diameter, a trial was placed on the HH, and a centering pin was inserted. Surface reaming prepared the implant bed, and periarticular osteophytes were removed to optimize ROM.

The labrum was separated from the glenoid, and the anterior and posterior vault margins were identified. Thirty-degree off-axis reaming allowed for glenoid preparation in conjunction with HH preservation. A guide pin was placed into the inferior aspect of the glenoid, the vault was prepared, the implant trialed, and pressurized cementation was used for final component placement. In patients with B2 glenoids, the biconcavity was corrected with debridement of the central ridge before placement of the guide pin and glenoid vault preparation.

After glenoid component placement, attention was redirected toward the HH. The fixation component was inserted into the center of the prepared socket, the humeral component was aligned with the superior edge of the supraspinatus insertion, proper morse taper connection was confirmed, and the implant was impacted. The subscapularis tendon was reapproximated, and the deltopectoral incision was repaired in the standard fashion.

### Rehabilitation

Postoperatively, patients were kept in a sling for 4 weeks. Passive ROM and pendulum exercises were initiated within the first week. Active and active assisted motion was initiated at 4-6 weeks with strengthening once full ROM is achieved. External rotation (ER) movement began after week 8. By 10-12 weeks postoperatively, moderate activity levels were allowed followed by graduated weight training with low-level isometrics, abduction, ER, and shoulder strengthening. Progressive strengthening programs with increased resistance began at 3-4 months postoperatively and included sport-specific training exercises for overhead activities dependent on milestone achievements in earlier phases. No activity restrictions were placed on patients after 4 to 6 months.

### Patient-reported outcomes

PROs included the American Shoulder and Elbow Surgeons Standardized Shoulder assessment form (ASES).^18^ A Visual Analog Scale for Pain (VAS-Pain) was used to measure the level of pain on a 10-cm line at the time of the evaluation. A score of 10 indicated the highest pain, and a score of 0 indicated no pain at all. An independent observer, not involved in preoperative patient selection and surgical treatment, measured active forward elevation (FE) and ER. Satisfaction was assessed with a numeric rating scale from 0 (very dissatisfied) to 10 (very satisfied).

### Radiographic analysis

Glenohumeral arthritis was staged on preoperative radiographic imaging according to the Samilson-Prieto Classification^20^ with a mild (grade 1), moderate (grade 2), or severe (grade 3) grade. All glenoids were assessed on axillary radiographs according to the original Walch classification.^29^ Radiographs were reviewed by three investigators, and consensus on the final classification was reached via Delphi majority method.^13^

Final AP and axillary radiographs were analyzed for periprosthetic radiolucency and component failure defined as loss of humeral taper connection, implant dislocation, glenoid fracture, and glenoid component dislocation.

**Figure 2:**
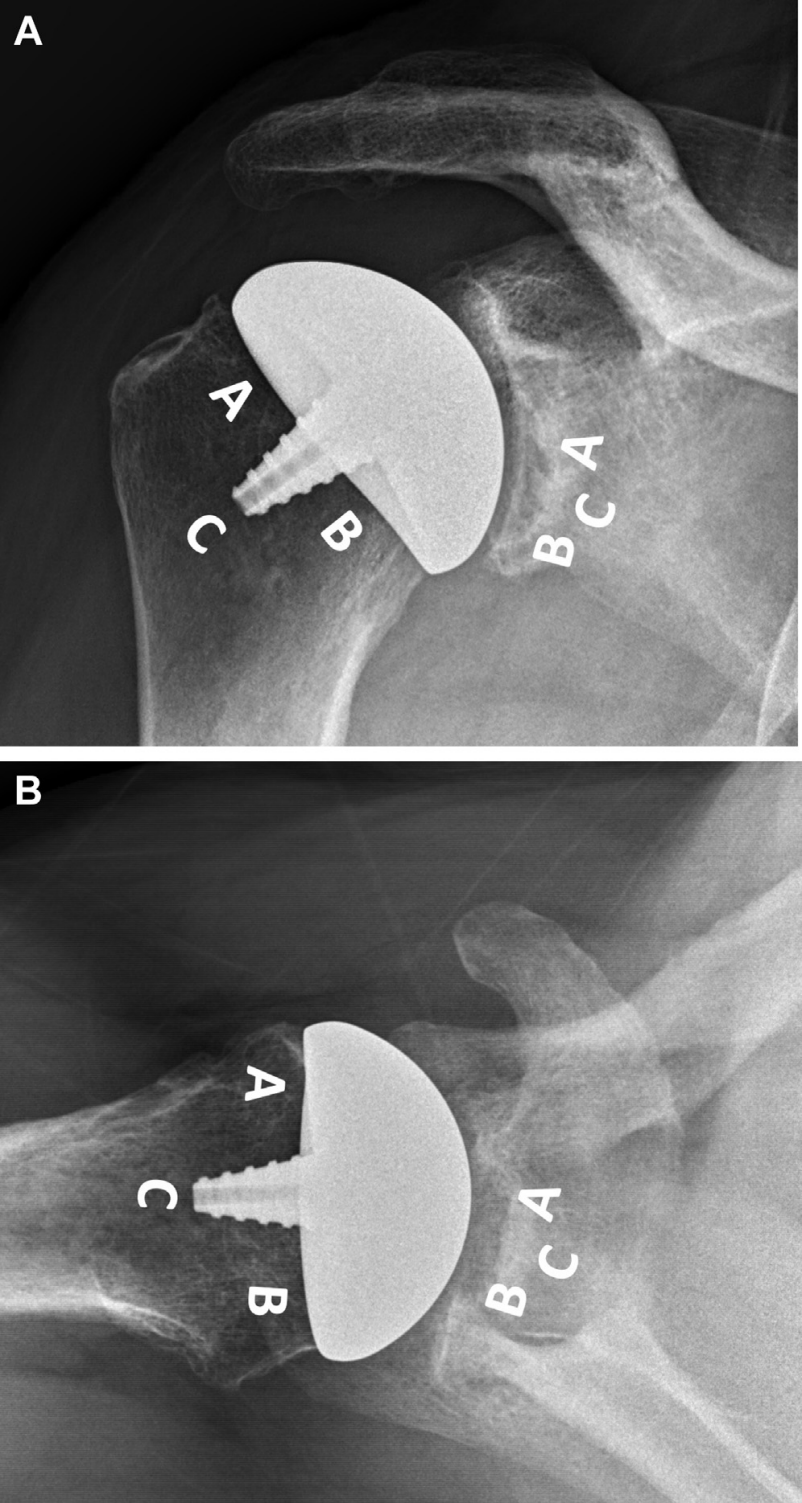
Assessment of periprosthetic radiolucent lines. (**A**) AP view. Humeral component: Zone A (superior undersurface), Zone B (inferior undersurface), Zone C (central fixation). Glenoid component: Zone A (superior undersurface), Zone B (inferior undersurface), Zone C (central fixation). (**B**) Axillary view. Humeral component: Zone A (anterior undersurface), Zone B (posterior undersurface), Zone C (central fixation). Glenoid component: Zone A (anterior undersurface), Zone B (posterior undersurface), Zone C (central fixation).

The risk of radiographic loosening was determined according to the study by Sanchez-Sotelo et al,^21^ who defined a threshold of 2 mm or greater observed in 3 zones or more. Glenohumeral components were divided into 3 zones: On AP imaging, zone A represented the superior undersurface, zone B the inferior, and zone C the area surrounding the humeral fixation component or glenoid peg. On axillary imaging, zone A represented the anterior undersurface, zone B posterior, and zone C as described for AP imaging (Fig. 2 *A* and *B*).

### Statistical analysis

#### Descriptive repeated measure analysis

Continuous data were reported as means and standard deviations, and categorical data as frequencies or percentages. Depending on the normal distribution, t-test or nonparametric methods were used to compare means. A Chi-square or Fisher’s exact test was performed to find differences in proportions. Where applicable, 95% confidence intervals were calculated.

A generalized linear mixed model was used, and the mean difference between preoperative and the last follow-up scores was compared considering the six postoperative follow-up measurements for each patient. A statistical analysis was performed using IBM SPSS software Version 27 (IBM Corp., Armonk, NY, USA). Statistical significance was set at the 5% level (*P* < .05).

#### Sensitivity analysis

Several studies established the minimal clinically important difference (MCID) and substantial clinical benefit (SCB) for PROs and ROM after total shoulder arthroplasty^23^^,^^24^^,^^30^ to assess patient-centric improvement. On the ASES score, Simovitch et al reported the MCID as a change from baseline by 17.0 and the SCB as a change of 37.6 at 50 months of follow-up. VAS-Pain MCID changes were 2.7, and SCB changes 3.8. MCID improvement for FE was 23^°^, and 46^°^ for SCB. On ER, MCID was established as an improvement of 15^°^, and 20^°^ for SCB.^23^^,^^24^ A sensitivity analysis was performed to determine the percent of patients who met or exceeded these thresholds (responder) on ASES, VAS-Pain scores, and ROM. In a secondary analysis, patients were grouped into low, medium, and high preoperative ROM to determine the effect on MCID and SCB improvements. ER groups included 0 to 20^°^, 21^°^ to 40^°^, and 41^°^ to 60^°^. The FE groups ranged from 60^°^ to 100^°^, 101^°^ to 140^°^, and >140^°^.

## Results

Thirty-nine shoulders in 36 patients (three bilateral) with a mean age of 65.9 years (range: 41-81 years) were included in this study (26 males and 13 females). The contralateral side in bilateral patients was treated at a mean follow-up of 18 months (range: 3-33 months) after the index procedure. The diagnosis was primary glenohumeral arthritis in all patients. The last follow-up assessment was performed at a mean of 41.0 months (range: 24-77 months) (Table I).

**Table I:** Preoperative patient characteristics for inlay total shoulder arthroplasty.

Description	Data
Number of shoulders	39 (3 bilateral)
Age (range), yr	65.9 (41-81)
Gender, male, n (%)	26 (66.7)
Gender, female, n (%)	13 (33.3)
Preoperative diagnosis, n (%) Osteoarthritis	39 (100)
Mean follow-up, (range), mo	41 (24-77)
Walch Classification, n (%)	
A1	10 (25.6%)
A2	10 (25.6%)
B1	8 (20.5%)
B2	10 (25.6%)
C	1 (2.6%)
Samilson Prieto Classification, n (%)	
Grade 1	0 (0%)
Grade 2	2 (5%)
Grade 3	37 (95%)

All patients were treated with a 20-mm glenoid component. Humeral component sizes extended across the entire range of HH implant dimensions. Six patients had a concomitant procedure which included three biceps tenodeses, two concomitant rotator cuff repairs, and one conversion from a partial to full head resurfacing seven years after the index procedure. No routine biceps tenotomies were performed.

The median operative time was 123 minutes (mean = 132, range = 75-300), and the median blood loss was 80 ml (mean = 102, range = 30-600). The procedure duration in one patient was 300 minutes with a blood loss of 600 ml due to massive musculature in a competitive weight lifter requiring extensive releases. No patient required blood transfusion, and all were treated on an outpatient basis with a hospital discharge under 23 hours. No reoperations or readmissions within 90 days were reported. One elderly female patient sustained a small posterior rim fracture during the index procedure and developed a fragment nonunion. At the last follow-up (3 years), her symptoms were acceptable negating the need for revision surgery to date. There were no other intraoperative or perioperative complications. One patient developed arthrofibrosis which was treated with arthroscopic lysis of adhesions at two years after surgery. No implant revisions were performed during the study period.

**Figure 3:**
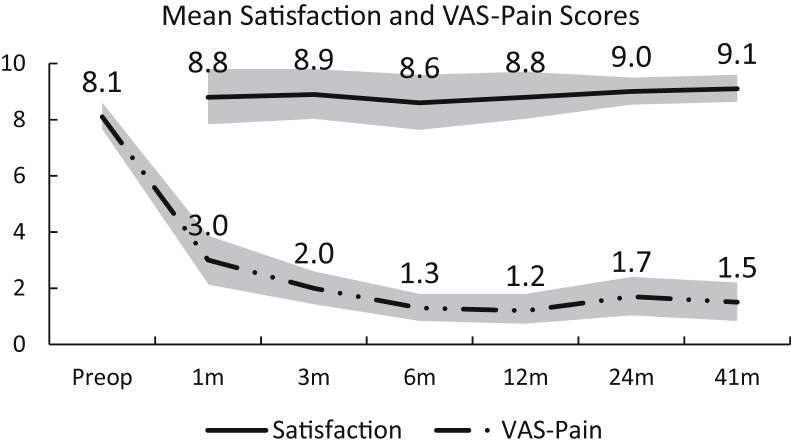
Pain and satisfaction assessment.

The ASES and VAS-Pain scores improved significantly, starting at one month postoperatively, and maintained their significance levels at all time points (*P* < .001) (Table II). The mean ASES score increased from 29.9 at baseline to 77.1 at the last follow-up, and the results were statistically significant (*P* < .0001). In post-hoc analysis, the mean difference between baseline and last follow-up ASES scores was 47.2 (Fig. 3*A*). Repeated measures analysis showed that the mean VAS-Pain score decreased from 8.1 at baseline to 1.5 at the last follow-up, and the results were statistically significant (*P* < .0001). In post-hoc analysis, the mean difference between baseline and last follow-up was -6.7 (Fig. 3*B*). The mean patient satisfaction rating was high throughout the follow-up and was rated best at the last visit (Fig. 3*C*) (Table II). The mean satisfaction score at 1 month postoperatively was 8.6 compared to 9.3 at the last follow-up, and it was statistically nonsignificant (*P* > .05). FE improved from 107° to 155° at the last follow-up, and ER improved from 23° to 51°.

**Table II:** Summary of patient-reported outcomes.

Outcomes	Preoperative	1 mo	3 mo	6 mo	12 mo	24 mo	41 mo
ASES							
Mean	30.4	52.1	72.1	79.4	84.0	78.2	77.1
SD	18.4	19.7	17.4	11.3	16.3	19.6	21.1
CI	24.6,36.2	45.3, 58.9	65.9,78.0	75.4,83.4	78.4,89.6	72.1,84.4	70.5,83.7
*P* value		<.001	<.001	<.001	<.001	<.001	<.001
VAS-Pain							
Mean	8.1	3.0	2.0	1.3	1.2	1.7	1.5
SD	1.7	2.5	1.8	1.4	1.6	2.1	2.2
CI	7.6,8.6	2.1,3.9	1.4,2.6	0.8,1.8	0.7,1.8	1.0, 2.4	0.8,2.2
*P* value		<.001	<.001	<.001	<.001	<.001	<.001
Satisfaction							
Mean	-	8.8	8.9	8.6	8.8	9.0	9.1
SD	-	2.8	2.6	2.8	2.5	1.7	1.6
CI	-	7.8,9.8	8.0,9.8	7.6,9.6	8.0,9.7	8.5,9.5	8.6,9.6

*ASES*, American Shoulder and Elbow Surgeons score; *SD*, standard deviation; *CI*, confidence interval; *VAS-Pain*, a pain visual analog scale.

**Figure 4:**
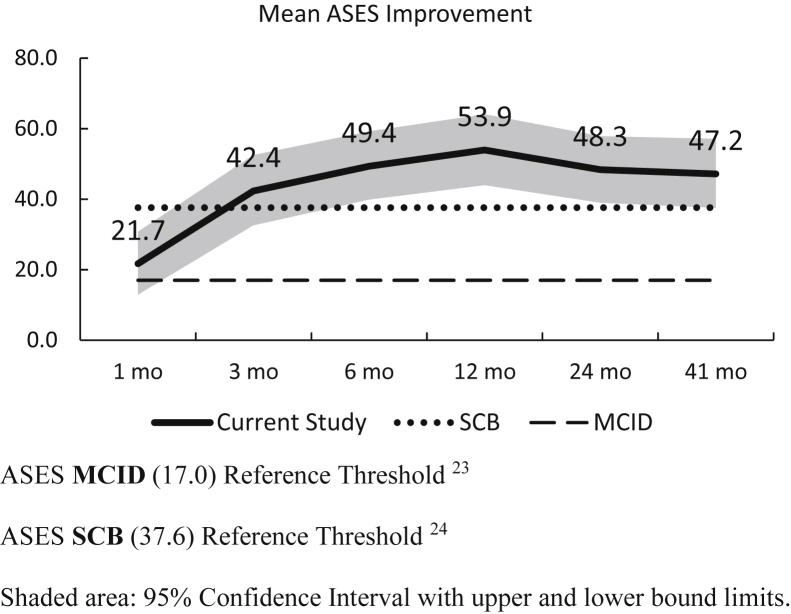
ASES scores. ASES MCID (17.0) Reference Threshold.^23^ ASES SCB (37.6) Reference Threshold.^24^ Shaded area: 95% confidence interval with *upper* and *lower* bound limits.

The average improvement in the ASES score was above the literature-reported MCID^23^ for total shoulder arthroplasty at one month postoperatively, at three months for the SCB,^24^ and stayed above both thresholds at all consecutive time points (Fig. 4).

**Figure 5:**
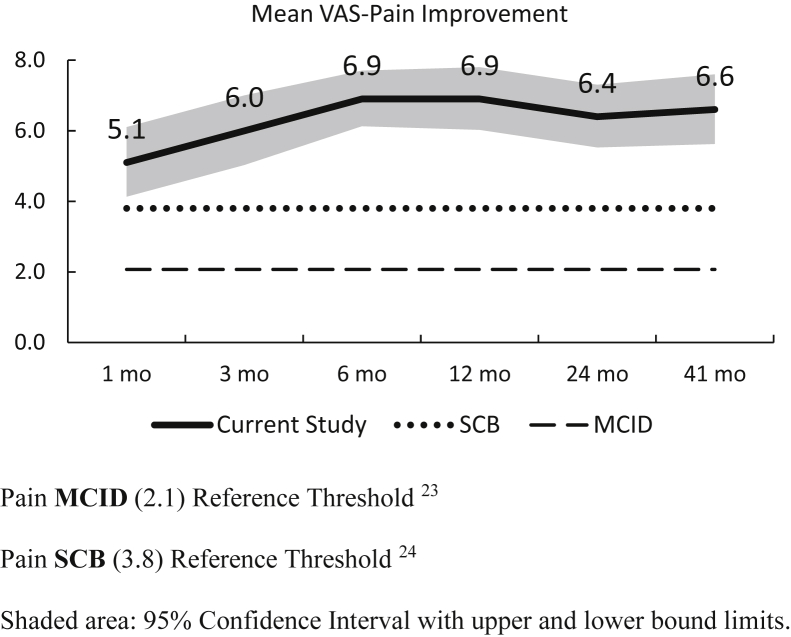
VAS-Pain improvement. Pain MCID (2.1) Reference Threshold.^23^ Pain SCB (3.8) Reference Threshold.^24^ Shaded area: 95% confidence interval with *upper* and *lower* bound limits.

**Figure 6:**
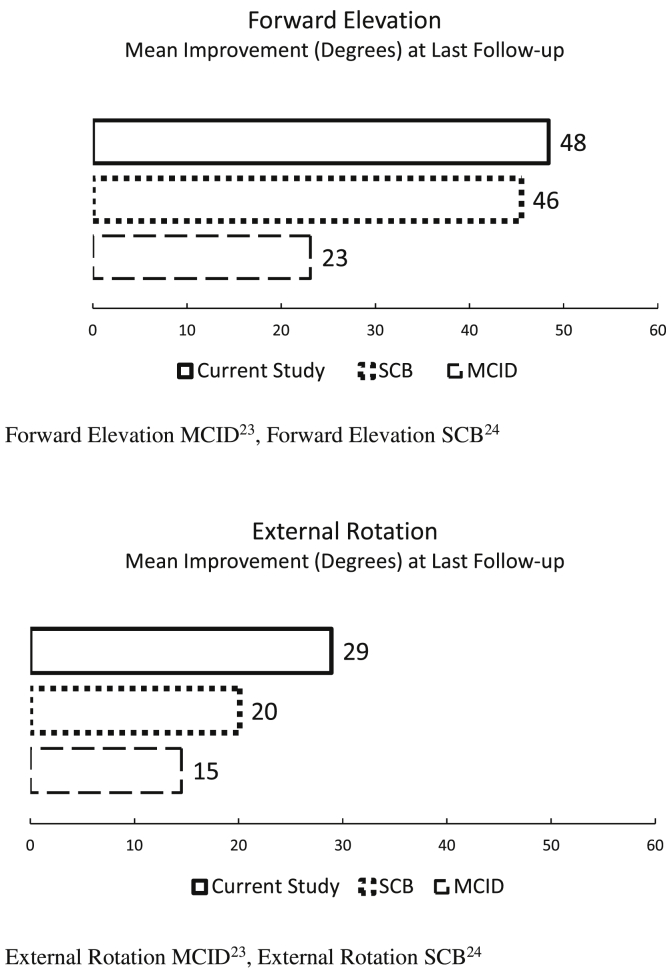
Mean improvement in forward elevation and external rotation. Forward elevation MCID,^23^ forward elevation SCB.^24^ External rotation MCID,^23^ external rotation SCB.^24^

The improvement in the mean VAS-Pain score was above both thresholds^23^, ^24^ at all postoperative time points (Fig. 5) as well as ROM improvement at the last follow-up (Fig. 6).

At the final assessment, 94.3% patients met or exceeded the ASES MCID^23^ indicating a “better” result, and 85.7% were responders for the SCB^24^ indicating a “much better” result. On VAS-Pain, 87.1% met or exceeded MCID, and 84.6% SCB. Improvement in ROM corresponded to 78.9% of patients meeting or exceeding MCID and 65.8% SCB levels on ER, and 76.9% (MCID) and 64.1% (SCB) on FE.

All three preoperative ROM groups were within 13° for ER (47.5° – 60°) and 10° for FE (151° – 161°) at the last follow-up (Table III). Patient improvement in the low group surpassed MCID and SCB on FE and ER, the medium group met MCID only, and patients in the high preoperative ROM group met neither.

**Table III:** Improvement in range of motion by preoperative group.

ROM	Preoperative group	Group range (degrees)	Mean improvement (degrees)	MCID^55^	SCB^54^	Group mean, absolute (degrees)	Group size (n/%)
External rotation	High	41-60	13.3	14.5	20.1	60.0	6/15.4
	Medium	21-40	15.4	14.5	20.1	47.5	12/30.8
	Low	0-20	**42.5**	14.5	20.1	50.0	21/53.8
Forward elevation	High	>140	3.9	23.1	45.5	161	9/23.1
	Medium	101-140	40.0	23.1	45.5	161	7/17.9
	Low	60-100	**68.3**	23.1	45.5	151	23/59.0

*ROM*, range of motion; *MCID*, minimal clinically important difference; *SCB*, substantial clinical benefit.

In bold, greatest improvement in patients with highest preoperative limitations.

Preoperative radiographic staging showed moderate to severe shoulder arthritis in all patients (Table I). All patients were assessed radiographically at the time of their clinical follow-up (mean: 41 months). None of the shoulders showed >2 mm of humeral periprosthetic radiolucency in any zone, and one patient sustained a glenoid rim fracture intraoperatively and developed progressive glenoid loosening of >2 mm. No other patients showed risks of implant loosening in 3 zones according to Sanchez-Sotelo et al.^21^

## Discussion

Treatment of advanced glenohumeral arthritis with iTSA resulted in high patient satisfaction, excellent pain relief, and functional improvement at an average follow-up of 41 months. No implants were revised during this study, and secondary procedures were limited to one shoulder requiring arthroscopic lysis of adhesions at 2 years.

Our goal was to determine absolute and relative improvements on PRO and ROM and assess postoperative changes using various sensitivity analyses. Werner et al established MCID thresholds at 24 months of follow-up,^30^ and Simovitch et al quantified MCID^23^ and SCB^24^ improvements at a mean follow-up of 49.7 months after stemmed TSA. Both thresholds were anchored on patient satisfaction questions allowing for a patient-centric measure of improvement across various outcomes metrics. Previous reports on iTSA showed significant improvement in ROM and function outcomes but did not interpret outcomes in response to these benchmarks.^4^^,^^6^

Patients in our series reported substantial pain relief at one month after the procedure and continued to improve in the first 6 months postoperatively. The lower bound of the confidence intervals for mean VAS-Pain scores were above both thresholds at all time points providing further support for meaningful postoperative pain relief. At the last follow-up, pain scores were comparable to results reported by stemmed TSA ranging from 1.3 to 2.1.^3^^,^^15^^,^^22^ VAS-Pain responder rates at 41 months were similar for MCID (current study: 87.1%, Simovitch et al^23^: 88.9%) and higher for SCB (current study: 84.6%, Simovitch et al^24^: 71.6%).

The lower bound of the confidence interval for mean ASES scores was above the MCID threshold^23^ starting at three months postoperatively, and above or at SCB levels^24^ from 6 to 41 months. ASES responder rates at 24 months for MCID (91.4%) and SCB (77.1%) were higher than those reported by Werner et al (78.2%, 70.0).^30^ Similarly, at the last follow-up, MCID (94.3%) and SCB (85.7%) responder rates compared favorably to those reported by Simovitch et al at 50 months (MCID: 92.7%, SCB: 79.5).^23^^,^^24^

Improvement in ROM was highly dependent on preoperative levels. Patients with substantial preoperative ROM deficits showed the highest improvement surpassing both thresholds at the last follow-up, whereas midrange ROM met MCID levels and high-range preoperative ROM groups reached a ceiling effect precluding them from any threshold improvements. Patients should be counseled accordingly when managing their expectations.

Our study reconfirmed two recent reports using nonspherical HH shapes coupled with inlay glenoid replacement. Egger et al^6^ compared their results of concentric vs. nonconcentric glenoids at 42.6 months of follow-up. The study found no significant differences between Walch Type A and Type B glenoids^29^ for the Penn Shoulder Score, ROM, and VAS-Pain. Cvetanovich et al^4^ found significantly improved clinical outcomes and no reoperations or radiographic loosening in an active population at 40.4 months of follow-up with a high rate of return to heavy occupational demand levels and sporting activities. The average ROM across all three studies exceeded 150° of FE and 50° of ER (current study: FE = 155° and ER = 51°). Recent systematic reviews summarized the results for various implant types: Uy et al reported on stemmed cemented TSA (pooled mean FE: 132°, ER: 37°) vs. pressfit implants (pooled mean FE: 146°, ER: 53°).^27^ Liu et al compared stemless TSA vs. stemmed TSA and reported similar postoperative functional outcomes and complication rates as well as a shorter operative time and decreased intraoperative blood loss for stemless implants.^17^ The weighted mean postoperative flexion for stemless implants was 142°, and 47° on ER.^17^ Ericson et al reported results of short-stem TSA in their recent systematic review and found a postoperative weighted average for flexion of 147°, and 48° for ER.^7^

There is a paucity of ROM data on total onlay resurfacing in the literature due to technical challenges associated with perpendicular onlay glenoid preparation and implantation. Levy and Copeland reported an improvement of FE from 68.0° – 128.0°,^16^ and Pritchett^19^ reported long-term FE of 119.0° and ER of 44.0°.

Our study reconfirmed a lower intraoperative blood loss with stemless implants: Patients in our series had an average blood loss of 102 ml compared to 496 ml in the stemless group reported by Liu et al and 593 ml in the stemmed group.^17^

Prior reports on inlay or inset glenoids were coupled with spherical HH replacement: Davis et al^5^ reported two-year results in 9 shoulders with advanced glenohumeral erosion and dysplasia showing a mean improvement on FE from 112.2° to 160.0° and from 27.8° to 41.7° on ER. Gunther and Tran^10^ reported the long-term results of 21 patients treated with inset glenoid coupled with spherical humeral replacement and showed an FE improvement from 95.0° to 131.0° and ER from 18.0° to 49.0°.

The risk of intraoperative periprosthetic fractures for stemmed implants has been reported with a wide range (1.2% to 16%)^1^^,^^28^ resulting from canal reaming, broaching, trial or implant insertion, retractor torque, and excessive ER.^8^ Significant risk factors have been associated with revision arthroplasty, female gender, and press-fit components.^1^^,^^28^

Postoperative periprosthetic humeral fractures have been reported after traumatic events or as a result of cortical weakening due to bone resorption, osteolysis, and stress riser effects from prosthetic loosening.^8^ Short-stem, canal sparing stemless and resurfacing implants may offer advantages mitigating some of these risks and move periprosthetic fracture patterns more toward the proximal humerus.^8^ In our series, no intraoperative or postoperative humeral fractures occurred, and no significant radiolucent lines were observed. Compared to short-stem and stemless designs, HH preservation combined with a short, threaded post used in our study may offer additional benefits against atraumatic or traumatic events.

More than two decades ago, Harryman et al^12^ investigated the effects of stemmed TSA and onlay glenoid replacement and demonstrated a net lateral humeral shift ranging from 5.5 mm to 7.0 mm resulting in a substantial tightening of the rotator cuff and joint capsule. Recent reports from the Australian Shoulder Arthroplasty Registry confirm these findings identifying soft-tissue disturbances as the primary reason for the revision of stemmed TSA.^2^

More recently, Gagliano et al^9^ compared the loading characteristics of onlay glenoids with a spherical humeral component to inlay glenoids with a nonspherical HH component. Onlay glenoid testing resulted in gross loosening of all components at a mean of 1126 cycles (range 747-1838), and none of the inlays showed gross loosening at the study endpoint of 4000 cycles. Combined glenoid rim (20.9N) and implant edge (73.3N) contact forces for inlay specimens were similar (94.2N) to that of the native glenoid rim (91.8N), whereas onlay glenoid specimens showed a 45% higher force at the implant edge (124.8N vs 85.7N). The authors concluded that the inlay glenoid showed superior biomechanical stability and resistance to loosening.^9^

Spherical HH implants dominate the current shoulder arthroplasty market despite growing evidence of a better fit^14^ and biomechanical advantages of nonspherical shapes. Hammond et al^11^ compared glenohumeral contact mechanics of spherical hemiarthroplasty with inlay HH resurfacing. The authors concluded that contoured HH resurfacing restored the geometric center of the HH better than hemiarthroplasty, with less eccentric glenoid loading which may limit glenoid wear and allow for better function. Jun et al^14^ assessed glenohumeral joint mechanics comparing native cadaveric specimen to randomly assigned nonspherical and spherical prosthetic heads. The nonspherical implant shape replicated the natural head shape, rotational ROM, and glenohumeral joint kinematics more accurately than the spherical prosthetic.

The strengths of this study included prospective follow-up of patients treated with a single TSA procedure allowing for longitudinal assessment of clinical results. Patients demonstrated increasing patient satisfaction over time with narrowing confidence intervals from 6 to 41 months of follow-up combined with excellent PRO improvement. Complications were limited to one patient developing arthrofibrosis which was treated with arthroscopic lysis of adhesions at two years postoperatively, and one patient with a small nonunion glenoid rim fracture who was managed conservatively. Off-axis glenoid preparation allows for glenoid vault access in conjunction with HH preservation; however, proper pin placement and high reamer speed before surface contact are important to avoid glenoid complications, particularly in elderly female patients.

The study was limited in cohort size and shorter term follow-up of 41 months, but our initial results demonstrated positive trends within this period. Literature-based MCID and SCB thresholds allowed for a suitable mechanism for an improvement-based sensitivity analysis. However, external satisfaction anchor questions, prosthesis selection, and follow-up make the comparison less generalizable. Future studies will need to establish these thresholds specifically for iTSA to reconfirm our results with internal satisfaction anchors. The study was further limited by selection bias, as all patients underwent the same procedure, regardless of the preoperative osteoarthritic and glenoid stage. Our study included a mix of Walch A1, A2, B1, and B2 glenoids.^29^ We used inlay glenoid resurfacing if the glenoid vault supported component placement. Ongoing enrollment, new follow-up, and further analysis of our prospective investigation will allow us to report on larger cohorts in the future and will make further subgroup analysis, including glenoid stage-specific results more meaningful.

Additional studies will need to determine if maintenance of glenoid version and joint line preservation combined with nonspherical HH implants provide a lasting improvement over current trends using stemmed procedures with advanced augmentation and reconstruction techniques and reverse TSA utilization in increasingly younger patients.

## Conclusion

Anatomic total shoulder arthroplasty using nonspherical HH and inlay glenoid replacement demonstrated significant improvement in PROs, excellent functional recovery, and patient satisfaction at short- to mid-term follow-up for the treatment of advanced glenohumeral arthritis with various glenoid stages. Clinical results provided supporting evidence on a bone-preserving low-risk option in primary shoulder replacement.

## References

[1] Athwal G.S., Sperling J.W., Rispoli D.M., Cofield R.H. (2009). Periprosthetic humeral fractures during shoulder arthroplasty. J Bone Joint Surg Am.

[2] Australian Orthopedic Association National Joint Replacement Registry (2020). Annual report 2020. https://aoanjrr.sahmri.com/annual-reports-2020.

[3] Bartelt R., Sperling J.W., Schleck C.D., Cofield R.H. (2011). Shoulder arthroplasty in patients aged fifty-five years or younger with osteoarthritis. J Shoulder Elbow Surg.

[4] Cvetanovich G.L., Naylor A.J., O'Brien M.C., Waterman B.R., Garcia G.H., Nicholson G.P. (2020). Anatomic total shoulder arthroplasty with an inlay glenoid component: clinical outcomes and return to activity. J Shoulder Elbow Surg.

[5] Davis D.E., Acevedo D., Williams A., Williams G. (2016). Total shoulder arthroplasty using an inlay mini-glenoid component for glenoid deficiency: a 2-year follow-up of 9 shoulders in 7 patients. J Shoulder Elbow Surg.

[6] Egger A.C., Peterson J., Jones M.H., Miniaci A. (2019). Total shoulder arthroplasty with nonspherical humeral head and inlay glenoid replacement: clinical results comparing concentric and nonconcentric glenoid stages in primary shoulder arthritis. JSES Open Access.

[7] Erickson B.J., Chalmers P.N., Denard P.J., Gobezie R., Romeo A.A., Lederman E.S. (2020). Current state of short-stem implants in total shoulder arthroplasty: a systematic review of the literature. JSES Int.

[8] Fram B., Elder A., Namdari S. (2019). Periprosthetic humeral fractures in shoulder arthroplasty. JBJS Rev.

[9] Gagliano J.R., Helms S.M., Colbath G.P., Przestrzelski B.T., Hawkins R.J., DesJardins J.D. (2017). A comparison of onlay versus inlay glenoid component loosening in total shoulder arthroplasty. J Shoulder Elbow Surg.

[10] Gunther S.B., Tran S.K. (2019). Long-term follow-up of total shoulder replacement surgery with inset glenoid implants for arthritis with deficient bone. J Shoulder Elbow Surg.

[11] Hammond G., Tibone J.E., McGarry M.H., Jun B.J., Lee T.Q. (2012). Biomechanical comparison of anatomic humeral head resurfacing and hemiarthroplasty in functional glenohumeral positions. J Bone Joint Surg Am.

[12] Harryman D.T., Sidles J.A., Harris S.L., Lippitt S.B., Matsen F.A. (1995). The effect of articular conformity and the size of the humeral head component on laxity and motion after glenohumeral arthroplasty. A study in cadavera. J Bone Joint Surg Am.

[13] Hsu C., Sanford B. (2007). The Delphi technique: Making Sense of consensus. Pract Assess Res Eval.

[14] Jun B.J., Lee T.Q., McGarry M.H., Quigley R.J., Shin S.J., Iannotti J.P. (2016). The effects of prosthetic humeral head shape on glenohumeral joint kinematics during humeral axial rotation in total shoulder arthroplasty. J Shoulder Elbow Surg.

[15] Levy J.C., Berglund D., Vakharia R., Tahal D.S., Mijc D., DeVito P. (2019). Midterm results of anatomic total shoulder arthroplasty with a third-generation implant. J Shoulder Elbow Surg.

[16] Levy O., Copeland S.A. (2004). Cementless surface replacement arthroplasty (Copeland CSRA) for osteoarthritis of the shoulder. J Shoulder Elbow Surg.

[17] Liu E.Y., Kord D., Horner N.S., Leroux T., Alolabi B., Khan M. (2020). Stemless anatomic total shoulder arthroplasty: a systematic review and meta-analysis. J Shoulder Elbow Surg.

[18] Michener L.A., McClure P.W., Sennett B.J. (2002). American Shoulder and Elbow Surgeons Standardized shoulder assessment form, patient self-report section: reliability, validity, and responsiveness. J Shoulder Elbow Surg.

[19] Pritchett J.W. (2011). Long-term results and patient satisfaction after shoulder resurfacing. J Shoulder Elbow Surg.

[20] Samilson R.L., Prieto V. (1983). Dislocation arthropathy of the shoulder. J Bone Joint Surg Am.

[21] Sanchez-Sotelo J., O'Driscoll S.W., Torchia M.E., Cofield R.H., Rowland C.M. (2001). Radiographic assessment of cemented humeral components in shoulder arthroplasty. J Shoulder Elbow Surg.

[22] Schoch B., Schleck C., Cofield R.H., Sperling J.W. (2015). Shoulder arthroplasty in patients younger than 50 years: minimum 20-year follow-up. J Shoulder Elbow Surg.

[23] Simovitch R., Flurin P.H., Wright T., Zuckerman J.D., Roche C.P. (2018). Quantifying success after total shoulder arthroplasty: the minimal clinically important difference. J Shoulder Elbow Surg.

[24] Simovitch R., Flurin P.H., Wright T., Zuckerman J.D., Roche C.P. (2018). Quantifying success after total shoulder arthroplasty: the substantial clinical benefit. J Shoulder Elbow Surg.

[25] Sweet S.J., Takara T., Ho L., Tibone J.E. (2015). Primary partial humeral head resurfacing: outcomes with the HemiCAP implant. Am J Sports Med.

[26] Uribe J.W., Botto-van Bemden A. (2009). Partial humeral head resurfacing for osteonecrosis. J Shoulder Elbow Surg.

[27] Uy M., Wang J., Horner N.S., Bedi A., Leroux T., Alolabi B. (2019). Cemented humeral stem versus press-fit humeral stem in total shoulder arthroplasty: a systematic review and meta-analysis. Bone Joint J.

[28] Wagner E.R., Houdek M.T., Elhassan B.T., Sanchez-Sotelo J., Cofield R.H., Sperling J.W. (2015). What are risk factors for intraoperative humerus fractures during revision reverse shoulder arthroplasty and Do they Influence outcomes?. Clin Orthop Relat Res.

[29] Walch G., Badet R., Boulahia A., Khoury A. (1999). Morphologic study of the glenoid in primary glenohumeral osteoarthritis. J Arthroplasty.

[30] Werner B.C., Chang B., Nguyen J.T., Dines D.M., Gulotta L.V. (2016). What change in American Shoulder and Elbow Surgeons score represents a clinically important change after shoulder arthroplasty?. Clin Orthop Relat Res.

